# Sex Clubs in the UK: Recreational Sex, Erotic Diversity and Geographies of Desire

**DOI:** 10.1007/s41978-022-00108-8

**Published:** 2022-06-09

**Authors:** Chris Haywood

**Affiliations:** Media, Culture and Heritage, School of Arts and cultures, Newcastle University, NE1 7RU Newcastle-upon-Tyne, UK

**Keywords:** Sex clubs, Recreational sex, Heterosexuality, Swingers, Leisure sex, Tourism

## Abstract

Current research has suggested that sex, sexual practices and sexual identities are increasingly being folded into people’s leisure and recreational activities. One area that has witnessed growing popularity has been sex clubs that market themselves as places that enable heterosexual casual, anonymous sexual encounters. Traditionally called swingers’ clubs, these are not strip clubs, lap dancing clubs or brothels, we have very little information about sex clubs or the people who visit them. In response, this article defines what sex clubs are, their geographical locations, and their facilities. Alongside this, through the data scraping of 6837 profiles of people who have visited clubs and left online reviews of the clubs that they have visited, this research provides the most extensive dataset available on the gender, age, relationship status and sexual preferences of sex club patrons. The findings from the study suggest that sex clubs are an emerging space for leisure sex that prioritises erotic practices that stand outside heteronormative norms and values. Whilst clubs have been traditionally associated with swinger communities, the findings in this article also suggest that sex clubs appeal to people with diverse sexual preferences. Alongside this, it highlights the potential ways in which sex clubs may be part of a broader spatialization of leisure sex. The article concludes by suggesting that in a post-Covid context, sex clubs will have increasing importance as places of leisure sex.

**Supplementary information:**

The online version contains supplementary material available at 10.1007/s41978-022-00108-8.

## Introduction

Across the UK, there are over forty-one sex clubs that provide opportunities for men and women to experience a range of sexual pleasures. These are not strip clubs, lap dancing clubs, gay/lesbian bars or sex entertainment venues; rather, they are often simplistically referred to in the popular media as ‘Swingers’ Clubs’ or ‘Swap Clubs’, providing a collective sex environment for ‘male-female couples having sex with other individuals and/or couples as part of their relationship’ (Byrne and Watts, 2011, p.81). Furthermore, these are not clubs where sex workers are knowingly employed or working; they are venues that men and women spend their leisure time visiting and often engaging in frequently anonymous sexual encounters. Estimates reported in the media suggest that sex clubs across the UK have one million visitors each year (Channel 4, 2020). Despite the proliferation of sex clubs in the UK, their increasing contribution to the economy and their role in an emergent culture of recreational sex and the mainstreaming of leisure and sex more generally (Attwood, 2014; Paasonen, 2016), we know very little about them. Whilst media accounts tend to focus on attention-grabbing headlines, academics, in contrast, have tended to be preoccupied with consensual non-monogamy (CNM) and how sex clubs provide a context for how those relationships are lived out. Not only do both accounts underplay the diversity of recreational sex practices in sex clubs; they tell us very little about the clubs themselves, where they are located, who visits them and what kinds of sex take place in sex clubs.

It is argued that sex clubs have emerged as part of a broader trend where “spaces and places for the purchase of sexual services and the commodities which facilitate sex as forms of leisure are increasing (Attwood, 2005; Smith, 2007)” (Attwood & Smith, 2013, 326). Furthermore, the imbrication of sex and leisure is providing a range of ways for the innovation and exploration of a range of lifestyles and individual identities (Berdychevsky and Carr, 2022; McCormack et al. 2021). This article contributes to this theoretical approach by understanding how sex clubs are commercializing new avenues for recreational sex, how they are providing spaces where sexual identities and cultures are played out and practiced and how clubs can be understood as part of a wider geography of leisure sex. These questions are part of a broader concern with sex clubs marketed at heterosexuals and an exploration of how sex clubs are both at the margins of the everyday order and yet be fundamentally a part of it. With a popular media that is episodically preoccupied with concerns about sex club legality, undercover exposes, and ribald headlines such as ‘*Outrage over swinger club in tiny village*’, *‘Sex club secrets revealed – five kinky things that REALLY go on behind closed doors’* or ‘*Willy Bonkas; Swingers club runs choc-themed orgy’*, the research aims to understand how these clubs are spaces that both disrupt and reinforce contemporary sexual norms and values.

One of the initial challenges of this project was to define what is meant by ‘sex club’. Academic research has tended to define sex clubs based on the sexual identities of those that visit them, such as swingers clubs (Gould, 2000), BDSM (Bondage, Domination and Sado-Masochism) and fetish clubs (Plante, 2006); Gay and Lesbian clubs (Escoffier and Meunier, 2019; Kitaka 1999). In contrast, Frank (2019) advocates for the use of ‘collective sex environments’ to identify commonalities between places and avoid a reductionism based on sexual identity or sexual practice (see also Garcia et al., 2015). However, in the popular media the term ‘sex club’ has been used to refer to a range of collective sex environments both in the UK and internationally that include strip bars, exotic dancing, privately organized sex parties, private orgies in hotel rooms, hired premises for events and meets, weekends away and holiday escapes, swinger conventions or festivals and caravan meets, franchised parties and events that tour the country, and saunas and spas that have rooms for sexual encounters. As a consequence, this creates difficulty when trying to disaggregate contexts and practices. In response, this article proposes a working definition of sex clubs that enables a more precise understanding of specific how leisure sex processes. This definition is based upon: clubs that describe their *primary* business as providing opportunities for on-premises heterosexual recreational sex; they are clubs whose premises are permanently situated in one location; they are clubs that are accessible to the general public and that clubs are explicitly run as a commercial venture. This criteria was thus operationalized as a key mechanism to identify and locate sex clubs and also as a means of understanding who visits them.

One of the reasons that we know very little about UK sex clubs is the inherent challenges that underpin researching such places. Many clubs market themselves on discretion and anonymity, a strategy that is mainly driven by an awareness of the social intolerance that surrounds non-normative sexual practices. More specifically, as O’Rourke (2005, p.11) has argued, ‘The lines of demarcation are so strict that anything which crosses over the monogamous, familial line is deemed to be deviant, pathological, illicit, culturally unintelligible’. Housed in factory units, renovated pubs or reclaimed retail premises, clubs are barely recognizable. By day, sex clubs might be mistaken for low-quality hotels, recently closed pubs or factory lock-ups; their anonymity crucially deployed as a form of cultural camouflage (Maines, 2001). However, by night, clubs become transformed into what Delph (1979) refers to as ‘erotic oases’: places for ‘edgy sex’ or ‘sexual behaviours and activities that might be considered to be at the borders or the edges of the permissible, desirable or conceivable’ (Pheonix and Oerton, 2013, p.163). Such sex might include but is not limited to anonymous sex, group sex, partner swapping, bukkake, same-sex practices, cuckolding, threesomes, ‘bareback sex’, gang bangs, BDSM, inter-racial and inter-generational sex. Sex often takes place in a range of ‘playrooms’, including but not limited to cinema rooms, dark rooms, couples-only rooms, saunas, private rooms, dungeons, glory hole rooms, grope rooms, orgy rooms, school rooms or mirror rooms. Some clubs have cars (and even a truck cab) to cater for the dogging experience. In one club, there is a tent with a light that projects the shadows of its users onto the wall next to it. These rooms often mimic classic pornscapes with highly theatrical, faux-grandiose interior designs swathed in reds, blacks and purples, with wipe-clean furnishings.

Whilst much work has been carried out documenting the histories of Gay and Lesbian clubs (especially in the US) (see Meunier & Escoffier 2021; Sides, 2006). there is very little literature on sex clubs that are marketed to heterosexuals. This article focuses on clubs marketed to heterosexuals because such (in)visibility of these sex clubs connects with cultural narratives of heterosexuality that position it as natural and ‘asexual’ (Phillips, 2006, 167; Richardson 1996). By focusing on clubs that are marketed to heterosexuals, the aim is to expose them to critical scrutiny, make them visible and understand the dynamics of these clubs as an emerging part of a leisure sex economy. Part of their (in)visibility means that it is unclear when and where sex clubs, as legal, commercial enterprises specifically designed to facilitate on-premises consensual sexual encounters for men and women, began to emerge. Roberts (2003) argues that sex clubs in the UK had their origins within informal swinger networks whose members were no longer satisfied with running meetings within their own homes and had gained experience from the existence of clubs dedicated to swingers in Europe. Roberts (2003) suggests that swinger clubs emerged as semi-professional enterprises that formalized leisure practices into a more organized practice. From the 1990s onwards, sex clubs in the form of warehouses and factory units began to spring up; these were much larger than the house parties and local bars where swinging parties were usually held. This shift in sex club provision can be understood as one from voluntarism within a subculture into an entrepreneurial commercial practice (Roberts, 2003). To put this into context, in 2003, there were no more than five on-premises sex clubs. In 2022, there are at least forty-one clubs operating across the UK. Although the onset of the COVID-19 pandemic signalled the closing of a number of clubs, it appears that post-Covid, a number of new clubs have emerged alongside the relaunching of more established clubs. This growth in the number of recreational sex clubs should be understood as part of a broader array of recently emerging sexual leisure opportunities. The increasing availability of sex clubs appears to support Attwood and Smith’s (2013, pp.334–335) suggestion that a new “…‘recreational sexuality’ underpinned by ‘self-pleasure and fun’, is becoming part of an ‘ethical retooling’ of consumer capitalism”.

The development of clubs in the UK has also been shaped by their ambiguous relationship to the law. Historically, clubs were subject to a range of acts, including the Disorderly Houses Act of 1751, which made it illegal to keep a ‘bawdy house’ where inappropriate sexual activity would take place, for example, brothels, adultery and fornication. At present, definitions of what counts as a sex entertainment is differentially applied by local authorities with some clubs are licenced to be sex entertainment venues, whilst others are not (Charalambides and Holland 2021). While there are some clubs that do provide forms of entertainment – for example, lessons in BDSM or burlesque entertainment – most clubs in the UK are registered as private members’ clubs. Alongside their ambiguous legal status, sex club applications for planning permission also provides little definitional clarity. In the UK, sex clubs tend to operate through seven diverse planning use categories as defined by the General Permitted Development Order (1995/2015), more commonly known as the Town and Country Planning Act. Some clubs operate under the same permissions as cafés, offices and hotels. Others operate under the same permissions as creches, law courts and places of worship. Yet others operate outside these categories and are based on residential usage. Furthermore, there are a large number of clubs for which planning applications are publicly unavailable. The implication here is that permission was granted prior to 1994 or even that some clubs have no planning permissions; a number of clubs have had their planning applications refused but continue to operate regardless of their planning status.

### Sex clubs and current research

Another reason for our lack of knowledge and understanding of sex clubs is the epistemological approaches of current research. For example, sex club premises have been used as a recruitment device to research men and women engaging in consensual non-monogamy (CNM). The overriding message of this research is that clubs operate as a backdrop to understanding such relationships. As a result, clubs have been instrumental to researchers exploring CNM and levels of self-esteem (Ruzansky & Harrison, 2019; Pugliese, 2019); CNM and identity narratives (Byrne and Haines, 2019); CNM and quality of parenting (Avanthay Strus, 2019); the demography, attitudes and practices of those involved in CNM (Wilt et al., 2018); and CNM and partner satisfaction (Kimberly, 2019). At times, sex clubs have been discussed as a feature of swinger lifestyles and how club attendance becomes a particular characteristic of swinger identities (Dukers-Muijrers et al., 2017; Heil et al., 2018). Thus, although sex clubs are mentioned in such studies, they operate primarily as the context for pursuing research questions that prioritize CNM.

Sex clubs have also featured in research examining sexual health risks. Work has been undertaken on sex clubs and their role in the transmission of STIs and HIV (Plateau et al., 2017; van Liere et al., 2013; Spauwen et al., 2018). However, sex clubs and sexual health risks have also primarily been explored in the context of couples engaging in CNM. One of the most comprehensive discussions in this area is provided by O’Byrne & Watts (2011) and their research in sex clubs on swingers and STIs. Along with making two observational visits to clubs and distributing a survey, they were able to collect in-depth demographic data on 72 individuals (38 men and 34 women). Alongside this, Niekamp et al. 2010) provide a more detailed analysis of how sex clubs contribute to the transmission of sexual infections. Rather than simply adopting a one-mode network approach that positions the individual at the centre of risk, a two-mode approach focuses also on the role of the venue in determining risk. As a result, Niekamp’s work provides an important shift from understanding sexual risks in CNM as an individual factor towards understanding risk in relation to the place of sex, frequency of attendance at the clubs, the kinds of sex practised in clubs and the role of drug use whilst in the venue (see also Spauwen, 2015; Evers et al., 2020).

The final area of research has focused on how sex clubs are an important dynamic in the shaping of CNM. The emphasis here is on how club dynamics impact upon how a consensual non-monogamous lifestyle is practised. Frank’s (2013) ground-breaking work on group sex provides one of the most in-depth explorations of swingers and the role of sex clubs in their relationships. She captures the range of rooms and the diversity of sex that takes place in different clubs and also how swingers in those spaces negotiate sexual encounters. She emphasizes how different locations can shape the nature of the sexual interactions that take place between lifestyles and the codes and etiquettes that are developed for couples to negotiate challenging scenarios. A similar focus is taken up by Bentzen & Træen (2014), who explore the sexual scripts of swingers within sex club settings. In their research, they specifically identify sex clubs as swingers’ clubs and explore how interpersonal relationships are managed and negotiated in club contexts. In their interviews with swingers recruited from online sites, they explore how club dynamics such as rules, time spent at the club and others attending can have an impact on how swinger relationships are affected by club dynamics (See also Kimberly and Hands, 2017; Harviainen & Frank 2016). Bentzen and Træen provide valuable accounts of the unspoken rules surrounding engaging or joining couples for sex. However, one of the limitations of this work is that sex clubs are often viewed as a context that is primarily dedicated to CNM and how their identities are lived out. In short, although details about clubs are provided, there is a tendency to treat them as spaces for CNM, with an implicit assumption that these are the only sexual communities that are visiting sex clubs.

## Methodology

Research on leisure sex appears to be making visible practices that have been relatively under-explored such as sex parties (Fulcher et al. 2018), chemsex (Hickson, 2018), geo-social networking apps (Cousineau et al. 2020) and men’s sex saunas (Jones, 2021). In a similar way, this article makes visible the sex clubs as leisure sex by providing systematically collected empirical data on 41 UK sex clubs, information gathered from over 6837 individual profiles of those who have visited sex clubs and left reviews of the club that they have visited. Although not discussed in this article, the data collected was complemented by ethnographic research conducted when visiting eighteen sex clubs in England. The aim of this methodological approach was simply to identify clubs, map their geographical locations and provide demographic data on those who visit them. The data collection for this article involved two phases.

The first phase involved collecting data from sex clubs across the UK. The definitional criteria outlined earlier in the article was operationalized and information was obtained from club’s online presence, which included their websites, records from Companies House and planning records. As a result of the application of the definitional criteria identified earlier in the article, between 2018 and 2019, there were forty-one sex clubs operating across the UK. The second stage of the research involved collecting a sample of profiles of people who had left reviews of sex clubs on a popular online sex-seeking site during 2018–2019. The research focused on clubs whose data was open-source (via Google). Even though participants had made this data public such data should always be reflexivity considered in order to protect participants (Nyck, 2022; Ravn et al., 2020). Because consent is not being clarified it is crucial that the data is presented in a responsible and ethically caring manner where de-identification and anonymization are employed robustly (Reed-Bernedt et al. 2022). Given the stigma that surrounds non-normative sexual practices, people who visit clubs and leave reviews generally seek anonymity. As a result, the research in this article has avoided using potential identifiers such as images, usernames, specific geographical locations and the clubs themselves. There are also ethical obligations to avoid negative effects towards the broader community who visit sex clubs. As a result, following a similar approach by Kingston & Smith (2020), the website and its address are not named. Therefore, there is no identifiable information that can link data to personal information.

A sample of 6837 publicly available individual profiles, including single women, single men, women in a coupled relationship, and men in a coupled relationship was initially collected. There were also a number of transvestites (TVs) and transsexuals (TSs) (terminology used by the site) who visited clubs and left reviews. The data collection process for the profile data involved using a team of associate researchers to manually scrape data. This mimicry approach to data scraping, where data is collected based on a predefined set of codes (Diouf et al., 2019), involved inputting data into an Excel spreadsheet. As a result, there was little scope for interpretive variance. The corpus was then cleaned to exclude multiple profiles and incomplete or unusable data (such as geographical location being described as ‘your bed’, or ‘town of pleasure’). As the data presented in this article was primarily pre-defined nominal data, reliability checks mainly consisted of systematically checking each entry across fifteen categories to ensure the consistency of cleaning. For example, where different clubs were reviewed by the same profile, duplicate profiles were removed. There were also 183 profiles that explicitly requested to be excluded from any research. From the profiles, data was collected on the club reviewers’ characteristics, including gender, sexual orientation, age group, ethnicity, geographic location and distance travelled to the club. Additionally, the data was sieved based on the definition of a sex club used in this article. Further information was collected on sexuality and sexual preferences; for couples, their sexual preferences relate to their combined interest as a couple rather than being disaggregated into the interests of the individuals. Furthermore, since the COVID-19 pandemic, reviews of clubs have been removed from the public and private spaces of the website. Ethical clearance for the research to take place was given by Newcastle University.

## Results

*Profile of Sex Clubs in the UK.*

The research findings (Table 1) suggest that the North West of England has the largest accumulation of clubs, clustered around cities such as Manchester, Bolton and Blackpool and towns such as Rochdale and Bury. In the South East and South West of England, clubs are far more disparate and spread out over wider areas. At the same time, the cost to enter clubs does not vary by region: the pricing structure is the same for all clubs with, on average, single men paying £30, couples £25 and single women and TV/TS paying £15.

**Table 1:** Number of sex clubs per UK region

East Midlands	3
East of England	3
London	3
North East	2
North West	11
Northern Ireland	0
Scotland	2
South East	3
South West	5
Wales	1
West Midlands	4
Yorkshire & Humber	4
**Grand Total**	**41**

Within the clubs themselves, where the data is available, there was a diversity of spaces available to engage in sexual practices (Table 2). Most spaces within clubs were playrooms. The number and themes of those playrooms varied from club to club. One club in the Midlands had at least nine different playrooms, consisting of themes such as a schoolroom, a hospital room and an Amsterdam room. Some of these clubs identified such playrooms as fetish rooms; in Table 2, these have been combined.

**Table 2:** Sex clubs’ facilities

	Licenced Bar	Female Glory Hole	Dark Room	Dungeon	Sauna	Playroom	Outside Area	Swimming Pool	Hot Tub
**East Midlands**	2	0	2	3	1	3	0	0	2
**East of England**	1	0	2	2	0	3	0	0	2
**London**	0	0	2	2	1	3	2	1	2
**North East**	1	0	2	2	2	2	0	0	2
**North West**	4	1	6	9	5	10	2	2	6
**Scotland**	0	0	1	2	0	2	0	0	6
**South East**	1	0	0	1	1	3	2	2	3
**South West**	0	0	2	3	1	5	1	1	3
**Wales**	0	0	1	1	1	1	0	0	1
**West Midlands**	1	1	3	3	1	4	1	0	2
**Yorkshire & Humber**	1	0	4	3	4	4	0	1	4
**Grand Total**	**11**	**2**	**25**	**31**	**17**	**40**	**8**	**7**	**33**

The range of spaces available for recreational sex is also demonstrated by the promotion of specific spaces such as the dungeon (primarily containing BDSM paraphernalia), and the dark room. The dark room normally involves a room that is often in darkness, leading to an increased sensation of anonymity. Out of the eighteen clubs visited, all had glory holes, although no club advertised this. In contrast, only two clubs in England had female glory holes. A female glory hole is usually a wall with an arched opening where the bottom half a female body becomes exposed. The top half of the body is out of sight and the space provides another form of anonymous sex. Whilst this data on spaces within the clubs provides a general overview, it should be added that some clubs would have a dungeon room even though they did not advertise it; other clubs would advertise a dark room, and this would be a small room with a three-quarter bed and an ultraviolet light. However, the data does provide an insight in the various spaces for recreational sex within the club.

## Club patrons: demographic data

**Table 3:** Sex, Relationship Status and Age of club visitors

Age Range	18–24	25–29	30–39	40–49	50–59	60–69	70+
**Male Single**	13	74	445	503	353	74	4
**Male MF Couple**	6	66	566	989	777	135	1
**Male MM Couple**	0	0	3	1	0	1	0
**TV/TS Single**	0	1	12	32	29	4	1
**TV/TS MF Couple**	0	0	0	2	0	0	0
**Female Single**	7	31	139	232	112	17	2
**Female MF Couple**	26	152	733	990	582	52	1
**Female FF Couple**	0	0	9	14	0	0	0

Two key issues emerge from the data in Table 3. In total, there are 4,366 reviews, left by 4,942 people in couples attending sex clubs and 1,895 people registered as single. The first finding in this data is that whilst couples are the majority grouping attending clubs often supported by dedicated couples’ nights, clubs are not simply couple-only spaces. Since 45% of all reviewer profiles are single, this has implications about those who are using clubs and the role of clubs in facilitating a range of heteroerotics that are not limited to a relatioanlly focussed CNM. Furthermore, the data indicates that single females make up 12% of those leaving reviews. In contrast, single men make up 31%. Those visiting sex clubs often refer to unattached females as ‘Unicorns’ to convey their rarity. It should be added that this is a partial picture. The experience of being in clubs, on nights that are not designated couple nights, suggests that the proportion of single men to couples and single females can often be more than two to one. A second finding from this data is that recreational sex within sex clubs is predominantly something for an older generation: the average age of women leaving reviews and attending clubs is 43 and the average age of men is 45. This has important implications, given how the popular media generally associates recreational sex with younger people.

The data on ethnicity presented in Table 4 is also distorted by the categories available on the website for club patrons to choose from. Providing an ethnic identifier on a profile is not required. As a result, the available categories are homogenizing in that self-selection does not reflect the diversity of the various ethnic categories.

**Table 4:** Club visitors by ethnicity

Ethnicity	Number	%
White	5142	71.5%
Black	136	1.9%
South Asian	36	0.5%
East Asian	8	0.1%
Mixed Race	32	0.5%
Latino	4	0.1%
Middle East	8	0.1%
Unknown	1825	25.4%

As a result, one-quarter of the reviewers did not provide information on their ethnicity. Again, it is important to be careful when reading this data. Clubs often promote racialized themed nights such as ‘Black Man’ Fan Club Nights’. On these nights, most attendees are black men, followed by white and black women. White men are the minority on such evenings. However, the majority of people visiting clubs and leaving reviews are white. Whilst Black men and women predominate over other ethnic minorities, there continues to be little understanding of the diversity of the ethnic heritage of participants who attend clubs.

### Sexual identities and sexual practices

Another aspect of the website where the affordance of the site shapes how people identify is around sexuality (Table 5). The sexualities listed on the profiles include straight, bi-curious, bisexual and gay. The most obvious absence is lesbian. This is not to suggest that lesbians do not visit sex clubs; club profiles suggest that two of the forty-one clubs hold events specifically for lesbians.

**Table 5:** Club visitors by sexuality

	Single Males	Single Females	MF Couple		TV	MM Couple	FF Couple
			Male	Female			
Total Sample Size (individuals)	1466	540	2540	2536	81	5	23
**Sexuality**	%	%	%	%	%	%	%
Straight	78%	28%	81%	19%	2%	0%	26%
Bi-Curious	12%	21%	11%	34%	11%	0%	9%
Gay	0%	0%	0%	0%	0%	50%	4%
Bisexual	10%	51%	8%	47%	87%	50%	61%
Unknown	0%	0%	0%	0%	0%	0%	0%

It is the online affordances that create the absence of lesbians in clubs, rather than their non-attendance. Furthermore, the data indicates a striking difference between the men and women who visit clubs and leave reviews. Around 76% of women identify as bisexual or bicurious, compared with 20% of men. It is suggested that the term bi-curious is used for those who are attracted to and have experienced or are considering having experiences with more than one gender (George, 2001; Ebin & Wagenen, 2006). There is a mixed picture of the prevalence of same-sex activity between men in heterosexual group sex and threesomes (Frank, 2008; Scoats, 2020). In this data presented here, it appears that men do not publicly identify with the identities associated with same-sex activities. However, it must be remembered that the online site where the data was collected is a dedicated site for heterosexual encounters; therefore, disclosure by men who desire an encounter that is not heteroerotic may affect their appeal to heterosexual couples and single women.

### Preferred sexual practices

It is not possible to disaggregate sexual interests at the individual level for those who are registered as couples. Although demographic data is available for individuals, data on sexual interests is collected on a couple-only basis. Table 6 provides an insight into the patterns of preferred sexual practices among those who attend clubs and leave online reviews. Swinging as an interest is not listed. However, same room swap and separate room swap, threesomes and group sex are the most popular activities that might point to swinging practices. Safe sex also has a high level of preference. The top five activities are all shared by men, women and couples, albeit in a different order. One of the biggest differences is between single men and MF couples; there is a 35% difference between men preferring dogging and separate room sex than MF couples. Between women and MF couples, the latter are much less keen on separate room swapping compared to single females. The biggest difference between men and women is dogging, with over 45% difference. In terms of TV/TS, the preferred practice of cross-dressing is around 80% with single men, single women and MF couples.

**Table 6:** Preferred sexual practices of those leaving reviews on clubs

	Overall	Male	Female	Couples
**Adult Parties**	91%	92%	85%	91%
**Anal**	43%	**60%**	30%	35%
**Blindfolds**	60%	58%	65%	60%
**Cross Dressing**	8%	9%	10%	5%
**Cuckolding**	35%	**57%**	21%	25%
**Cybersex**	13%	**23%**	5%	9%
**Dogging**	38%	**63%**	18%	28%
**DP**	48%	62%	35%	42%
**Fisting**	23%	**37%**	22%	15%
**Gangbangs**	51%	**70%**	38%	42%
**Group Sex**	81%	86%	68%	81%
**Phone Sex**	14%	**28%**	4%	8%
**Safe Sex**	90%	90%	90%	90%
**Same Room Swapping**	79%	76%	72%	83%
**Separate Room Swapping**	45%	65%	61%	**30%**
**SM**	23%	25%	28%	20%
**Spanking**	52%	54%	59%	49%
**Swingers’ Clubs**	93%	92%	91%	95%
**Threesomes**	91%	94%	85%	90%
**Toys**	76%	68%	77%	79%
**Watersports**	15%	**26%**	13%	9%
**Webcams**	22%	33%	7%	19%

Another interesting difference to emerge is that only 30% of couples indicate that separate room sex (where one partner leaves the other partner to have sex in a private room) is an interest, as opposed to 65% of men and 61% of women. The suggestion here is that single men and women have an interest in sex in a private rather than a public space. Same room sex is an interest for over 83% of couples, with similar levels of interest expressed by single men and women (76% and 72% respectively). This also has implications for the kind of sex that is sought within clubs. The most popular interest for females in relation to men and couples is blindfolding; the most popular sexual practice for men, as opposed to couples and women, is anal sex. Finally, safe sex is listed as an interest for 90% of single females, single males and couples. The implication of this is that based on the sample presented in this paper, 10% or 600 profiles do not list safe sex as an interest. We must be careful not to suggest that this group of people advocate non-safe sex, but simply that they have not listed safe sex as an interest. Further research is needed to understand why this sexual preference is not selected. Equally, we should not assume that those who have listed safe sex as an interest are all participating in safe sex. Ethnographic data provided elsewhere estimates that at least one in every four sexual encounters includes unsafe sex (Haywood, 2022).

### Geography and club visitors

Another dataset, Table 7, provides information on the demography of the geographical location of the reviewer. The dataset was mined to understand where visitors to clubs lived. Using the 48 counties of England, together with Scotland, Wales and Northern Ireland, we can identify that more people visiting clubs and writing reviews come from Greater Manchester, Lancashire, the West Midlands and Greater London. The lowest numbers of people visiting clubs and leaving reviews come from the Isle of Wight, Rutland, Herefordshire, Northern Ireland and Oxfordshire. However, the population sizes of the locations of people writing reviews of clubs have also been taken into account and we can see that proportionally, Lancashire, Cheshire, Greater Manchester, West Yorkshire and the West Midlands have visitors to clubs who leave the most reviews.

Table 7 also shows a sample of areas with the highest and lowest number of reviews, it also compares the number of reviews against the population data for each area. This table shows that the number of reviews cannot be explained solely by the size of the population in each region. It is particularly notable that the number of reviews made by reviewers from Greater London is low compared with the percentage of people living in Greater London. This could be explained either by the demography of the area, or that their visits to clubs are linked with patterns of working in London. In contrast, reviewers reporting their location as Lancashire leave a disproportionately high number of reviews. This could be explained by the fact that there is a high concentration of clubs in Lancashire and surrounding areas.

If we combine Table 6 with Table 7, it is possible to map out club visitor’s preferred sexual practices with the region that they live. It would appear that both Sussex and East Sussex eschew safe sex, with the lowest number of profiles expressing a preference for safe sex practices. With a combined total of 73% of profiles expressing a preference for safe sex, combined with a national average of 89%. Shropshire shows a prevalence for kinkier sex, with the highest percentage of profiles expressing a preference for engaging in S&M with 43% compared to a national average of 26%. The quest for kink continues with 38% (compared with 23% national average) of profiles from Shropshire also expressing a predilection for Fisting. Removing those results that are skewed due to the size of the population of profiles, fisting is also popular in both Northern Ireland and Gloucester, where 39% of respondent in both areas have expressed a preference for the practice. Both Lincolnshire and Cambridge, are the least keen in the country at 32% of profiles stating separate room swapping as an interest, compared to a 43% national average. Residents of Buckinghamshire seem content to play with their own partners showing the lowest preference for group sex (65% compared with a national average of 81% ), also coming in below average for an expressed preference to both same room swapping (74% against an average of 79%) and amongst the lowest expressed preference for gangbangs (32% against a national average of 49%). Interestingly, they expressed an average interest in threesomes at 88% (compared with a national average of 88%). Further work is needed to understand how the sexual preferences at a regional level implications for the prevalence of clubs may have, how they market themselves, their facilities and the thematizing of their evenings.

**Table 7:**
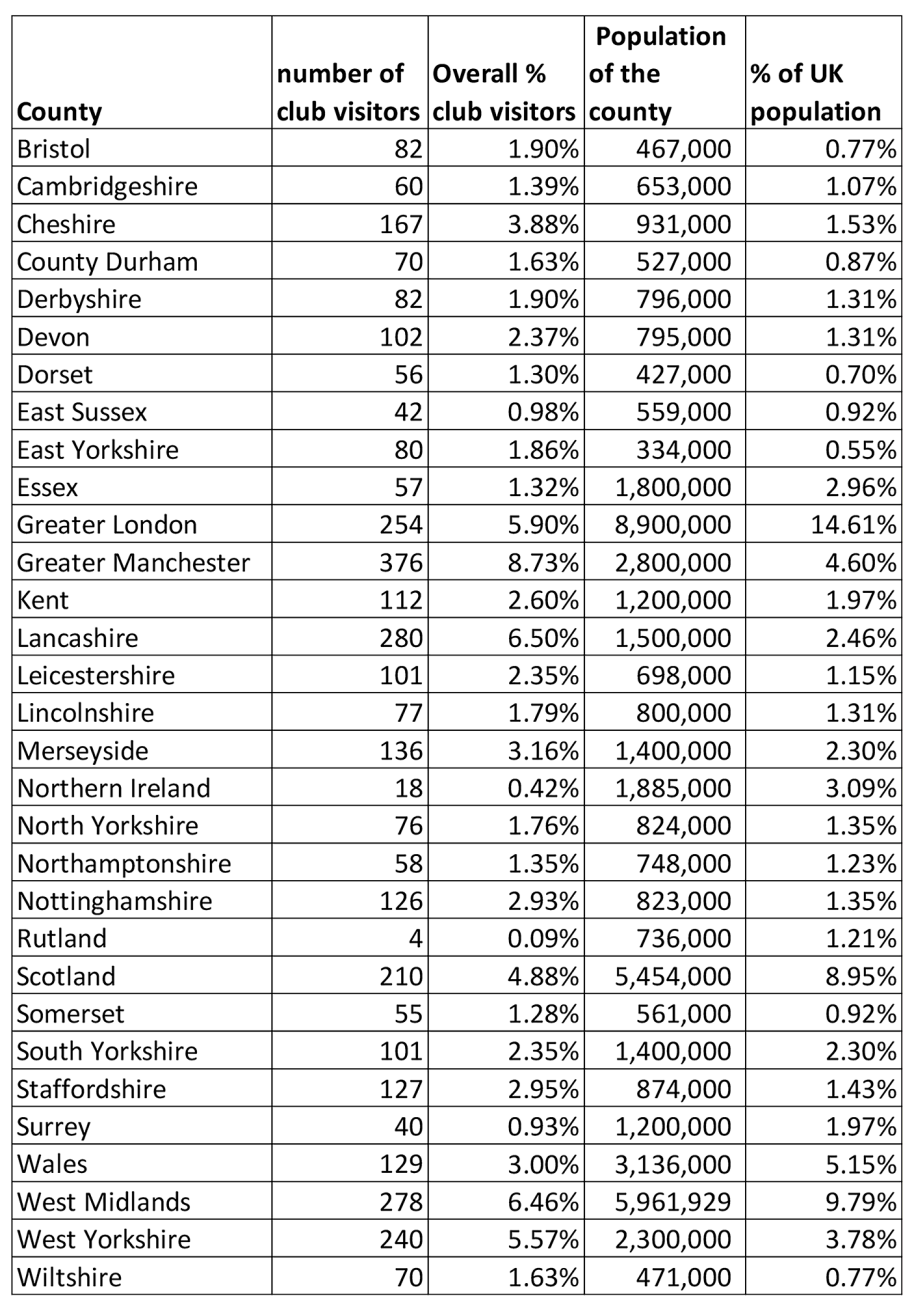
UK regions with the highest and lowest number of profiles that visited clubs

## Discussion

The increasing mainstreaming of sex appears to have resulted in the emergence of closer connections between sex and leisure. Whilst some leisure sex activities have become highly visible on UK high streets in the form of erotic retail, sex clubs continue to remain relatively undisturbed. Apart from the occasional newspaper exposes, television documentaries or dramas or celebrity or political scandals, discussions about sex clubs tend to be overshadowed by more contentious commercialized leisure sex such as strip-clubs, pole dancing and burlesque clubs and brothels. The aim of this article was to contribute to identifying how sex clubs are part of the commercialization of recreational sex, to understand more about those who visit clubs and to think about how sex clubs can help geographically locate leisure sex. The following discussion explores these aims further by focusing on how clubs facilitate recreational sex, promote erotic diversity and geographically place leisure sex.

### Sex clubs and recreational sex

The availability, accessibility of clubs and the range of their facilities (Tables 1 and 2) suggests that sex clubs are becoming an important space for recreational sex. It is argued that this growing sector of the sex economy points to more people in the UK using sex as a leisure activity or ‘leisure sex’ (Attwood & Smith, 2013; Berdychevsky & Carr, 2020). Often pitching themselves as ‘lifestyle clubs’, sex clubs provide a way of doing sex that is not dependent on monogamy, marriage and reproduction. This means that in contrast to relational sex, which refers ‘to building relationships, creating a family, or cementing committed relationships’, sex clubs enable the pursuit of the experience of sex itself, rather than the experience of being part of heteronormative relationships (Penhollow et al., 2010). It is argued that recreational sex signals a cultural shift in late modern society, where sexual values are becoming unanchored from traditional narratives of sex and love and instead are emerging as sexual lifestyles that are shaped by individualism, consumption and leisure (Attwood, 2011). More specifically, Attwood (2011, p.83) argues that ‘the emergence of late modern recreational sexuality is linked to – and can be seen as emblematic of – a broad range of contemporary concerns with image, lifestyle and self-exposure, which have become means of selfcare, self-pleasure and self-expression’. Furthermore, the sex club as a space for recreational sex resituates sexual pleasure outside of the domestic sphere into a public sphere, ‘generally eschewing the traditional view that sex should be a relationship enhancing and/or child-producing endeavor confined to monogamous, married, adult partners’ (Wright, 2012, p.120).

Being outside of the private sphere, different clubs promote themselves on the promise of the consumption of (unique) experiences (Table 2). These include having the longest swimming pool, the largest number of playrooms, or the opportunity to engage in unique erotic opportunities. The experience of the club has the elements of the fairground, where different spaces become places to experience different pleasures. Just like the theme park, ‘the primary focus…is on providing visitors with extraordinary, immersive and theatrical experiences of high emotional value (Lukas, 2008; Manthiou et al., 2016; Reiter, 2004)’ (Cabana, 2020, p.2). Similar to marketing practices within tourism (Benitez and Lopez, 2019), sex clubs attempt to thematize patron experiences by re-constructing popular cultural fantasies of the erotic through their playrooms. The re-creation of relationships between teacher/pupil, doctor/patient and prison officer/prisoner suggests that cultural narratives of the forbidden can, through fantasy, be lived out in the space of the sex club. The diversity of the resources offered by clubs also points to a possible conceptual difference between leisure and recreation. Whilst leisure has often been associated with timeframes (Mills, 2008), recreation with its traditional roots in restoration and recuperation offers a possibility of understanding sexual encounters that suggest a re-creation of the self (Stothart, 1998). It is important to recognize that this potential re-creation of self that is typically associated with younger people (Vinodrai, 2017; Wilson, 2019), is also being taken up by an older generation.

Whilst current discussions of recreational sex are predominantly concerned with technology-driven health and well-being risks to young people and broader society, the data in this research enlarges the scope of leisure sex. In the context of concerns about young people hooking-up and casual sex and an associated ‘sexualization’ issue related to Tinder/Grindr, the data suggests that leisure sex is not specific to younger people. Sex clubs are predominantly visited by older people (average 43 F/44 M) (Table 3). It is suggested that not only do sex clubs enable recreational sex to take place, but they are facilitating a recreational sexual practice that is primarily appealing to an older generation. One of the reasons for the increase in older people’s engagement with leisure sex, according to Freak-Poli (2019, p.27), is that ‘new generations of older adults are more extroverted, spend more time out of a marital relationship, are less ashamed of their sexual desires and engage in more sexual behaviour that is more varied’. The implication is that a generation that has experienced the collapse of traditional kinship ties are now engaging in leisure sex. Furthermore, corresponding with work on older people and sexual infections (Dukers-Muijrers et al., 2010; Tuddenham et al., 2016; Brown 2020), approximately 10% of those leaving reviews of clubs do not list safe sex as a sexual preference. More recently, in 2020 the majority of new HIV were in heterosexual communities and the majority of late diagnoses (where infection is already detrimental) was recorded in heterosexuals aged over 45 (Health Security Agency, 2021). More work needs to be undertaken to understand the potential public health implications of sex clubs, especially in relation to their emphasis on anonymity and discretion.

### Erotic diversity and the limits of swinging

If the previous section highlighted how sex clubs provide the possibilities of engaging in leisure sex in alternative places, clubs also enable the possibilities of new ways of practicing sex. Berdychevsky (2018, p. 11) points out that sexual leisure spaces can ‘serve as important playgrounds where sexual and gendered identities can be negotiated, contested, inverted, and transformed.’ It is argued that sex clubs can be seen as such a playground. Clubs offer a range of different sexual opportunities that involve de-coupling heterosexual erotics from heteronormativity. This supports Berlant and Warner’s (1998, p.548) claim that ‘Contexts that have little visible relation to sex practice, as life narrative and generational identity, can be heteronormative in this sense, while other contexts forms of sex between men and women might *not* be heteronormative. Heteronormativity is thus a concept distinct from heterosexuality.’ Alongside couples-only nights, clubs promote events that attend to a wide range of sexual preferences, such as inter-generational, inter-racial, group and multi-gendered sex. These are often marketed as Greedy Girls Night, Black Cock Fan Clubs, Young Guns and mums’, TV and TG (Transgender) admirers nights, BBW’s and Curvy Girls Specials, Gang Bang evenings and T-Girl Times. Thus, whilst the clubs accommodate and appeal to traditional swinger communities, they also offer a range of sexual opportunities that go beyond relationally focused CNM. Not only is there evidence that clubs are illustrative of leisure sex; recreational sex is enabling a range of different erotic possibilities to emerge. Furthermore, erotic diversity does not only relate to a disconnection between heteronormativity and heteroerotics, but this form of leisure sex is also primarily public in nature. The promotion of erotic diversity via the club architecture, such as exhibitionism, voyeurism, dogging suggests that a scopophilic dynamic underpins sexual encounters. Whilst there are rooms that are lockable or have curtains, erotic diversity is structured through the facilitation of a variety of sexual encounters. This diversity of erotic encounters can also be understood in relation to the sexual preference practices of those who visit clubs and leave reviews.

The data on sexual preferences of those reviewing and attending clubs also suggests that the definition of sex clubs as ‘Swinger’ or ‘Swap clubs’ may be too narrow. Those who visit clubs and leave reviews indicate a wide range of preferences that are not simply about swapping partners (Table 6). It appears that MF couples tend to disassociate from sexual practices that may be deemed more ‘hardcore’ such as anal, fisting, gang bangs and watersports; practices for which men have the most preference. Alongside this, the most preferred sexual practices among single women were blindfolds and spanking rather than S/M. These different preferences suggest that within the sex club context, there is the potential for a range of erotic diversity to take place. In turn, the range of preferred practices challenges pervasive understandings of traditional heterosexuality. One way to think about the range of sexual practices associated with heterosexuality is to consider clubs as queer places of heteroeroticism, where sexual practices take place that contest, replace and reconfigure what we understand as heterosexuality (Powell, 2019). However, it should be added that the subversive potential of the sex club appears to be taken up by clubgoers who are primarily white (Table 4). This opens up conversations about who is able to access and engage in different forms of recreational sex (Gill et al., 2018).

The argument being presented in this section is that sex clubs are more than just places for swingers to meet; they enable a wider range of heteroerotic practices to take place. Furthermore, sex clubs also facilitate sexual encounters beyond the heteroerotic. Despite clubs marketing themselves as places for heterosexuals, one of the most striking results to emerge from the research is that over 70% of women visiting clubs identify as bisexual or bi-curious (Table 5). Sex clubs appear to operate as a space where the display and practice of same-sex desire between women is enabled. Existing research tells us that the spaces for the expression of women’s bisexuality are extremely circumscribed by popular heteropatriarchal narratives: ‘the “bisexual woman” is constructed as a male heterosexual fantasy, rather than as an autonomous or oppositional erotic agent’ (White, 2008). The marketing of ‘bi nights’ represents women’s same-sex desire as exotic and interesting and resonates with Irigaray’s (1985, p.25) claim that women’s bodies are ’a more or less obliging prop for the enactment of man’s fantasies’. On the other hand, it is important to acknowledge and recognize that sex clubs are emerging as spaces that facilitate women with the opportunity to self-identify with bisexual identities and/or engage in bisexual practices. We need to be careful not to reduce women’s identifications to a simple appendage or consequence of heteropatriarchal desire and in turn, erase women’s agency. Instead, we need to understand how sex clubs appear to be part of a broader cultural shift where women’s desire is becoming enmeshed with sexual consumerism within emerging forms of leisure sex (Attwood & Smith, 2013; Illouz, 2017). This may (or may not) lead to ‘openings for considering sexuality as degrees of variation, experimentation and transformation’ (Paasonen, 2018, p.5). For example, in contrast to women’s identification with bisexual activity, only 15% of men identify with a bisexual desire. Whilst this corresponds to previous research within swinger communities (Frank, 2008), it complicates more recent work that suggests that more sex between men in threesomes is becoming more prevalent (Scoats et al. 2021).

Whilst this section has discussed how sex clubs facilitate a winder range of erotic practices and not simply as a conduit for swinger networks, the next section contextualizes sex clubs as part of a broader spatialization of leisure sex.

### Geographies of (P)leisure

One of the most challenging aspect of this research was to place sex clubs as leisure sex in the context of geography. Throughout the UK, various places have developed reputations as sex hotspots. A dogging layby in Surrey, the hook-up cultures in Blackpool, Gay cruising on Hampstead Heath, and the A1 as the ‘kinkiest road in Britain’ point to how sexual practices and places become imbricated. Sex clubs are enmeshed within this erotic topography (Bell, 1994), with clubs contributing to the UK’s sexual cultural imaginary and urban mythologies. Although the individual reviews provide a rich qualitative understanding of club experiences, the geographical data provides a broader insight into how leisure sex is being experienced across the UK. The majority of reviews posted by visitors primarily relate to clubs located in the North of England. As highlighted above, clubs in the North and in the Midlands tend to be found in clusters (Table 1) – sometimes with at least three within twelve miles of one another. One explanation for this could be that clubs in different locations adopt familiar repertories of public intimacy (McNair, 2003; Kaplan 2021). These repertories refer to the ways in which entertainment venues shape, configure and manage experiences within those spaces. For example, sex clubs in the North often take on the characteristics of a working-class club or pub. Part of the evening may involve playing bingo, singing karaoke or eating from a buffet. In contrast, some clubs in the Midlands tend to adopt a more ‘night club’ experience. This can sometimes involve a venue recreating the nightclub dance floor, DJ and long bar. Other areas such as Greater London and more rural locations appeal to a more bespoke ‘clientele’, with a more selective guest list and upmarket furnishings. These generalizations need to be unpacked further to test further the links between geography and place and how sex clubs are experienced. Alongside this, the data on the preferred sexual practice of those who visit clubs and leave reviews and where they live (Tables 6 and 7), suggests that more work needs to be done on the inter-relationships between the location of the club, the sexual preferences of the club patrons, where those patrons live and the club’s approach to public intimacy and its success.

A second insight from the data suggests that people in some counties and regions in the UK are more likely to visit sex clubs (Table 7). The data provides us with a snapshot to understand where people who visit clubs are travelling from. It is not surprising, given the clustering of clubs within the North West, that a quarter of all visitors leaving reviews come from this area. However, the figures suggest that more people from Greater Manchester, Lancashire and Cheshire are visiting and reviewing clubs than anywhere else in the UK. More work is required to understand how sex clubs are being integrated within these communities and why people from these areas are more likely to publicly review their visit. Chris Ryan’s (2000) early definition of sex tourism as ‘sexual intercourse while away from home’ helps to convey the idea that sex tourism may not be simply located in well-known cities such as Bangkok or Amsterdam. Furthermore, Frank (2003) adopts Urry’s (1990) concept of ‘touristic practices’ to explain visits to clubs by those practicing CNM in the USA. The uncoupling of sex tourism from exotic locations resituates the recreational nature of sexual encounters as something that frequently takes place in cities, towns and villages throughout the UK. Clubs themselves are beginning to connect with where they are situated with nearby hotels and in some cases ‘places to visit’ whilst in the area. At the same time, the relationships between the location of club visitors and their preferred sexual practices could provide another nuance. Given that there appears to be patterns of preferred sexual practices associated with certain geographical regions, there is the possibility of clubs facilitating (and of course commercializing) particular sexual cultures based on certain sexual preferences to develop. Overall, it could be argued that the emergence of sex clubs has led to what might be considered a ‘tourismification’; ‘a socio-economic and socio-cultural process by which society and its environment have been turned into spectacles, attractions, playgrounds, and consumption sites’ (Wang, 2000, p.197).

## Limitations

There are a number of limitations to the approach in this paper, most notably around the mining of club reviews for information. First, the review platforms for the clubs are voluntary: they rely on people who have visited the club to leave a review. It is difficult to predict the differences between those who visit clubs and leave reviews and those who don’t leave reviews. However, research suggests that people leave online reviews for a number of reasons. For example, it may be a result of altruism (Hoyer & van Straaten, 2021) or it may be a form of self-promotion, demonstrating a sense of credibility (Park et al., 2014). It is important to note that the profile data is based on those people who have visited the club and left a review. Thus the data is skewed, not only because the sample is based on people who have left reviews and are members of an online sex seeking community (people who attend clubs may not necessarily have an online profile), but also because many people who visit clubs do not necessarily leave a review. We also need to be careful about the veracity of profile data being entered. Given that attending clubs continues to be subject to a general social stigma, some of the data entered on the profiles may be deliberately misleading. For example, residential location may be cloaked in a way to avoid detection, leading to a more generalized location.

We also need to be careful about how profile data is read. The data being used is also skewed because the primary aim of joining a sex seeking community is often to meet people. Therefore, the profile may be constructed in a way that enhances the user’s self-expression (Shen et al., 2014). For example, it is apparent that men will tick numerous boxes in terms of interests, whereas women and couples appear far more selective. Also, sexual interests on the profile may have been constructed when a person first joined a sex seeking community; it is unclear how much profiles are changed and manipulated in order to ensure that they achieve more meets. Thus the aim is to present themselves as appealing to others rather than to provide an accurate picture of their sexual likes and dislikes. Therefore the data on sexual interests may also be, to some extent, biased. Research has highlighted that ‘daters’ self-presentation behaviours tend to be strategic and intentional’ (Tong et al., 2020), so that the information that is posted online tends to be a means to be noticed. At the same time, such information is always tempered by a need to demonstrate authenticity. This tension in self-presentation also provides a filter to how to interpret the profile data. Other work has highlighted that the less physically attractive someone assumes themselves to be, the more they will manipulate their age, weight and height to appear more favourable (Hancock et al., 2007).

## Conclusions

Although sex clubs in the UK frequently appear in popular culture such as news reports, television documentaries, films and music videos, there continues to be an emphasis on sexual restraint and inhibition; characteristics ‘deeply ingrained in the national character’ (Leach, 2004, p.133). As such, sex clubs, alongside a range of other forms of recreational sex, remain one of the UK’s ‘dirty secrets’. In many ways, this article has provided an insight into what constitutes a sex club, where they are located, who visits them and the sexual preferences of those who visit. Alongside this empirically driven exploration of sex clubs, the article has advocated an epistemological shift that moves away from viewing sex clubs as part of a broader ecosystem of CNM, and instead suggests that clubs have become the space for increasing erotic diversity. It should be added that this diversity is not simply a place of hedonistic erotic intensity where ‘anything goes’. Rather, with its emphasis on the scopophilic, sexual encounters in clubs are often hierarchal, where those with the most erotic capital (Green, 2014), such as women, black bodies and younger bodies, tend to be more desirable. There is thus a diversity of erotic practice, one albeit circumscribed by hierarchies of desire.

Although the impact of the COVID-19 pandemic may have accelerated the closure of some clubs, in a post-Covid context, there is evidence to suggest that sex clubs are thriving as a number of new clubs begin to emerge and existing clubs have held relaunch parties. Whilst much work continues to be done on what happened to sex during Covid, competing media narratives of the impact of the pandemic on post-Covid sexual intimacy are beginning to take shape. On the one hand, the post-Covid world is going to result in a momentous orgiastic ‘fuckfest’ (Christakis, 2020); on the other, the anxiety of forging new sexual intimacies is producing a longer-term disconnection that is disrupting the ways in which sexual connections are being made. Although much of the research for this article was undertaken prior to the impact of the pandemic, subsequent visits to the clubs, post-lockdown, suggest a more uncertain sexual intimacy. It is an intimacy that is driven by an ambiguity of when or if the opportunities for heterosex might be closed down, whilst there is also a need for people to visit clubs to experience a sexually intimate space without having sex. Either way, there is no doubt that sex clubs as places for recreational sex may even gain even more social, cultural and economic significance not simply as a result of the pandemic, but as an increasingly popular space for recreation and ‘touristic practices’.

## Electronic supplementary material

Below is the link to the electronic supplementary material.

Supplementary Material 1

## References

[CR2] Attwood F (2011). Sex and the citizens: Erotic play and the new leisure culture. The new politics of leisure and pleasure.

[CR3] Attwood, F. (Ed.). (2014). *Mainstreaming sex*. Bloomsbury Publishing

[CR4] Attwood, F., & Smith, C. (2013). Leisure sex: More sex! Better sex! Sex is fucking brilliant! Sex, sex, sex, SEX. *Routledge handbook of leisure studies* (pp. 347–358). Routledge

[CR5] Avanthay Strus, J. (2019). *Manitoban Consensual Non-monogamous Couples’ Conciliation of Their Parenting Role and Their Sexual Lifestyle During the Transition to Parenthood* (Doctoral dissertation, Université d’Ottawa/University of Ottawa)

[CR6] Bell D (1994). Erotic topographies: On the sexuality and space network. Antipode.

[CR7] Bentzen AS, Træen B (2014). Swinging in Norway in the context of sexual health. Sexuality & Culture.

[CR8] Berdychevsky L (2018). ‘Risky’ leisure research on sex and violence: Innovation, impact, and impediments. Leisure Sciences.

[CR9] Berdychevsky L, Carr N (2020). Innovation and impact of sex as leisure in research and practice: Introduction to the special issue. Leisure Sciences.

[CR10] Berlant L, Warner M (1998). Sex in public. Critical inquiry.

[CR11] Brown, D. (2020). Attitudes and Beliefs Related to Risk of Sexually Transmitted Infection in Swingers Who Do Not Use Condoms. *Walden Dissertations and Doctoral Studies*, 9066

[CR12] Cabanas E (2020). Experiencing designs and designing experiences: Emotions and theme parks from a symbolic interactionist perspective. Journal of Destination Marketing & Management.

[CR90] Charalambides, L. and Holland, C. (2021). No sex discussion please, we’re British. *Journal of Licensing,* 30, 4–10

[CR13] Christakis NA (2020). Apollo’s Arrow: The Profound and Enduring Impact of Coronavirus on the Way We Live.

[CR14] Cousineau LS, Johnson CW, Parry DC (2021). ‘What’s my score?’: the complexities of straight male Geo-Social Networking Application use. Leisure Studies.

[CR15] Dean T (2015). No Sex Please. We’re American. American Literary History.

[CR16] DeAndrea DC, Tong T, Liang S, Levine YJ, Walther JB (2012). When do people misrepresent themselves to others? The effects of social desirability, ground truth, and accountability on deceptive self-presentations. Journal of Communication.

[CR17] Delph E (1978). The silent community: Public homosexual encounters.

[CR18] Diouf, R., Sarr, E. N., Sall, O., & Birregah, B. (2019). Mamadou Bousso, and Sény Ndiaye Mbaye. “Web scraping: state-of-the-art and areas of application.“ In *2019 IEEE International Conference on Big Data (Big Data)*, pp. 6040–6042. IEEE,

[CR19] Dukers-Muijrers NH, Niekamp AM, Brouwers EE, Hoebe CJ (2010). Older and swinging; need to identify hidden and emerging risk groups at STI clinics. Sexually transmitted infections.

[CR20] Dukers-Muijrers NH, van Rooijen MS, Hogewoning A, van Liere GA, Steenbakkers M, Hoebe CJ (2017). Incidence of repeat testing and diagnoses of Chlamydia trachomatis and Neisseria gonorrhoea in swingers, homosexual and heterosexual men and women at two large Dutch STI clinics, 2006–2013. Sex Transm Infect.

[CR21] Ebin J, Wagenen AV (2006). Developing successful sexual health and support services for bisexual people: Lessons learned from the BiHealth program. Journal of Bisexuality.

[CR22] Evers YJ, Dukers-Muijrers NH, Kampman CJ, van Liere GA, Hautvast JL, Koedijk FD, Hoebe CJ (2020). Prevalence of drug use during sex among swingers and perceived benefits and risks–a cross-sectional internet survey in the Netherlands. Sexually transmitted infections.

[CR23] Frank K (2003). “Just trying to relax”: masculinity, masculinizing practices, and strip club regulars. Journal of Sex Research.

[CR24] Frank K (2008). Not Gay, but Not Homophobic: Male Sexuality and Homophobia in the Lifestyle. Sexualities.

[CR25] Frank, K. (2013). *Plays well in groups: A journey through the world of group sex*. Rowman & Littlefield Publishers

[CR26] Freak-Poli R (2020). It’s not age that prevents sexual activity later in life. Australasian journal on ageing.

[CR27] Fulcher K, Shumka L, Roth E, Lachowsky N (2019). Pleasure, risk perception and consent among group sex party attendees in a small Canadian Urban Centre. Culture, Health & Sexuality.

[CR28] Garcia JR, Escasa-Dorne MJ, Gray PB, Gesselman AN (2015). Individual differences in women’s salivary testosterone and estradiol following sexual activity in a nonlaboratory setting. International Journal of Sexual Health.

[CR29] Gould T (2000). The Lifestyle: A Look at the Erotic Rites of Swingers.

[CR30] George S (2001). Making sense of bisexual personal ads. Journal of Bisexuality.

[CR31] Gill, R. (2009). Beyond the sexualization of culture thesis: An intersectional analysis of sixpacks, midriffs and hot lesbians in advertising. *Sexualities*, *12*(2), 137–160

[CR32] Hancock, J. T., Toma, C., & Ellison, N. (2007, April). The truth about lying in online dating profiles. In *Proceedings of the SIGCHI conference on Human factors in computing systems*, 449–452

[CR33] Harviainen, J. T., & Frank, K. (2016). Group sex as play. *Games and Culture: A Journal of Interactive Media*

[CR1] Haywood, C. (2022). *Sex Club: Recreational Sex, Fantasies and Cultures of Desire*. Palgrave. Forthcoming.

[CR34] Health Security Agency. (2021). *HIV testing, new HIV diagnoses, outcomes and quality of care for people accessing HIV services: 2021 report*. DHSC

[CR35] Heil, J., Hoebe, C. J., van Loo, I. H., Cals, J. W., van Liere, G. A., & Dukers-Muijrers, N. H. (2018). Hepatitis E prevalence in a sexual high-risk population compared to the general population.PloS one, 13(1), e0191798 10.1371/journal.pone.0191798 PMC5784977 29370254

[CR36] Hickson F (2018). Chemsex as edgework: towards a sociological understanding. Sexual health.

[CR37] Hoyer, B., & van Straaten, D. (2021). *Anonymity and Self-Expression in Online Rating Systems-An Experimental Analysis* (70No. vol.). Paderborn University, Faculty of Business Administration and Economics

[CR38] Illouz, E. (Ed.). (2017). *Emotions as commodities: Capitalism, consumption and authenticity*. Routledge

[CR39] Irigaray L (1985). This sex which is not one.

[CR40] Kaplan D (2021). Public intimacy in social media: The mass audience as a third party. Media, Culture & Society.

[CR41] Jones, S. S. Y. (2021). Play by design: The porn-viewing room in Taiwanese and Korean men’s sex saunas.Sexualities,13634607211023850

[CR42] Kimberly C (2019). A measurement to assess transition, maintenance and satisfaction in the swinging lifestyle. Journal of Family Therapy.

[CR43] Kimberly C, Hans JD (2017). From fantasy to reality: A grounded theory of experiences in the swinging lifestyle. Archives of Sexual Behavior.

[CR44] Kingston, S., & Smith, N. (2020). Sex counts: An examination of sexual service advertisements in a UK online directory.The British Journal of Sociology 10.1111/1468-4446.12727 31903606

[CR45] Kitaka (1999). Kitaka’s Experiment or Why I Started the Ecstasy Lounge. Journal of Lesbian Studies.

[CR46] Leach, J. (2004). *British film*. Cambridge University Press

[CR47] Lukas, S. A. (2009). *Theme Park*. Reaktion books

[CR48] Maines, R. P. (2001). *The technology of orgasm: ‘Hysteria,’ the vibrator, and women’s sexual satisfaction* (24No. vol.). JHU Press

[CR49] Manthiou A, Kang J, Chiang L, Tang L (2016). Investigating the effects of memorable experiences: An extended model of script theory. Journal of Travel & Tourism Marketing.

[CR50] Meunier, É., & Escoffier, J. (2021). Gay Collective Sex in New York City from the Late 1800s to Today. *The Gayborhood: From Sexual Liberation to Cosmopolitan Spectacle*, 85

[CR51] Mill, R. C. (2008). The inter-relationships between leisure, recreation, tourism, and hospitality. *The SAGE handbook of hospitality management*, 90–106

[CR52] Niekamp AM, Mercken LA, Hoebe CJ, Dukers-Muijrers NH (2013). A sexual affiliation network of swingers, heterosexuals practicing risk behaviours that potentiate the spread of sexually transmitted infections: a two-mode approach. Social Networks.

[CR53] Nycyk, M. (2022). Ethical Use of Informant Internet Data: Scholarly Concerns and Conflicts.Journal of Digital Social Research,1–22

[CR54] O’Byrne P, Watts JA (2011). Exploring sexual networks: A pilot study of swingers’ sexual behaviour and health-care-seeking practices. CJNR (Canadian Journal of Nursing Research).

[CR55] O’Byrne, P., & Haines, M. (2019). A qualitative exploratory study of consensual non-monogamy: sexual scripts, stratifications and charmed circles.Social Theory & Health,1–18

[CR56] O’Rourke M (2005). On the eve of a queer-straight future: Notes toward an antinormative heteroerotic. Feminism & Psychology.

[CR57] Paasonen, S. (2016). Pornification and the Mainstreaming of Sex. In *Oxford Research Encyclopedia of Criminology and Criminal Justice*

[CR58] Park H, Xiang Z, Josiam B, Kim H (2014). Personal profile information as cues of credibility in online travel reviews. Anatolia.

[CR59] Penhollow, T. M., Marx, A., & Young, M. (2010). Impact of recreational sex on sexual satisfaction and leisure satisfaction.Electronic Journal of Human Sexuality, Vol.13

[CR60] Phillips R (2006). Unsexy geographies: Heterosexuality, respectability and the travellers’ aid society. ACME: An International Journal for Critical Geographies.

[CR61] Phoenix, J., & Oerton, S. (2013). *Illicit and illegal*. Routledge

[CR62] Plante RF (2006). Sexual spanking, the self, and the construction of deviance. Journal of Homosexuality.

[CR63] Platteau T, van Lankveld J, Ooms L, Florence E (2017). Sexual behavior and sexually transmitted infections among swingers: results from an online survey in Belgium. Journal of sex & marital therapy.

[CR64] Powell R (2019). Hardcore Style, Queer Heteroeroticism, and After Dark. Feminist Media Histories.

[CR65] Pugliese, S. B. (2019). *It and Them: Self-Esteem in Swing Lifestyle and Swingers* (Doctoral dissertation, Keiser University)

[CR66] Ravn S, Barnwell A, Barbosa Neves B (2020). What is “publicly available data”? Exploring blurred public–private boundaries and ethical practices through a case study on Instagram. Journal of empirical research on human research ethics.

[CR67] Reed-Berendt R, Dove ES, Pareek M, UK‐REACH Study Collaborative Group (2022). The Ethical Implications of Big Data Research in Public Health:“Big Data Ethics by Design” in the UK‐REACH Study. Ethics & human research.

[CR68] Reiter, A. (2004). The hybrid consumer of leisure squeezed between fun maximization, chill out, and the radical search for inner values. *The tourism and leisure industry: shaping the future*, 173–180

[CR69] Richardson, D. (Ed.). (1996). *Theorising heterosexuality: Telling it straight*. Open University Press

[CR70] Roberts, M. (2003). *Related to Bigotry: The Repression of Swingers in Early 21st century Britain* (53 vol.). Liberation Alliance. Sociological Notes

[CR71] Ruzansky AS, Harrison MA (2019). Swinging high or low? Measuring self-esteem in swingers. The Social Science Journal.

[CR72] Ryan, C. (2000). Sex tourism: paradigms of confusion? *Tourism and sex: culture, commerce and coercion*, 23–40

[CR73] Salas Benítez, C. M., & López López, Á. (2019). Spatial effects of cultural thematization for recreation and tourism in the pedestrian cultural corridors in Mexico City’s Historic Center. *Investigaciones geográficas*, (98)

[CR74] Scoats, R. (2020). *Understanding Threesomes*. Gender, Sex, and Consensual Non-Monogamy. Routledge. **)**

[CR75] Shen, W., Maceli, K., Zhao, Y., Baack, D. W., & Bacon, D. R. (2014). The impact of gender and national culture on electronic word of mouth communications. *Proceedings of the Association of Collegiate Marketing Education*, 145–161

[CR76] Sides J (2006). Excavating the postwar sex district in San Francisco. Journal of Urban History.

[CR77] Spauwen LW, Niekamp AM, Hoebe CJ, Dukers-Muijrers NH (2018). Do swingers self-identify as swingers when attending STI services for testing? A cross-sectional study. Sex Transm Infect.

[CR78] Spauwen LW, Niekamp AM, Hoebe CJ, Dukers-Muijrers NH (2015). Drug use, sexual risk behaviour and sexually transmitted infections among swingers: a cross-sectional study in the Netherlands. Sex Transm Infect.

[CR79] Stothart B (1998). Refining and defining: Contributions to ongoing debates. New Zealand Physical Educator.

[CR80] Tong ST, Corriero EF, Wibowo KA, Makki TW, Slatcher RB (2020). Self-presentation and impressions of personality through text-based online dating profiles: A lens model analysis. New Media & Society.

[CR81] Tuddenham SA, Page KR, Chaulk P, Lobe EB, Ghanem KG (2017). Patients fifty years and older attending two sexually transmitted disease clinics in Baltimore, Maryland. International journal of STD & AIDS.

[CR82] Urry J (1990). The tourist gaze: Leisure and travel in contemporary societies.

[CR83] van Liere GA, Hoebe CJ, Niekamp AM, Koedijk FD, Dukers-Muijrers NH (2013). Standard symptom- and sexual history–based testing misses anorectal Chlamydia trachomatis and Neisseria gonorrhoeae infections in swingers and men who have sex with men. Sexually transmitted diseases.

[CR84] Vinodrai, T. (2017). Planning for “cool”: Millennials and the innovation economy of cities. *The Millennial City* (pp. 27–37). Routledge

[CR85] Wang, N. (2000). *Tourism and modernity: A sociological analysis*, Pergamon (p. 197)

[CR86] Wilson PK (2019). Am I invisible?! Millennial invisibility in America. Journal of Ethnic and Cultural Studies.

[CR87] Wilt J, Harrison MA, Michael CS (2018). Attitudes and experiences of swinging couples. Psychology & Sexuality.

[CR88] White JD (2001). Bisexuals who kill: Hollywood’s bisexual crimewave, 1985–1998. Journal of Bisexuality.

[CR89] Wright, P. J. (2012). Is internet pornography consumption related to adult US males’ sexual attitudes.American Journal of Media Psychology, *5*(1–4), 118 – 28.

