# Supplementary material for: Sex Clubs in the UK: Recreational Sex, Erotic Diversity and Geographies of Desire
Source: Int J Sociol Leis. 2022 Jun 9;5(3):297–320. doi: 10.1007/s41978-022-00108-8 (PMC9178535; doi:10.1007/s41978-022-00108-8)
Supplement: Supplementary file 1 — Supplementary Material 1 [file 41978_2022_108_MOESM1_ESM.docx]

Manuscript number: IJSL-D-21-00021

Title: Sex clubs in the UK: Recreational sex, erotic diversity and geographies of desire

I genuinely appreciate the time that the reviewers have taken to look at this piece of work. Reviewing is always a time-consuming process and there is an obligation as an author to repay this time and effort by taking the comments seriously and, where possible, engaging and integrating them as much as I can. The comments have pushed me to make clearer what this article is actually doing. So in response to the comments, I have read over the article several times with a critical eye not only to address the specific points raised but to embed those points where relevant into the rest of the article.

|  | Rev. |  |  |
| --- | --- | --- | --- |
| 1 | 2 | Reviewer #2: P 7 L 58 & 2 other - NCM - should this be CNM? Or something else, it is not clear | I have identified instances of the different acronyms being used and made them consistent (and explained it)! |
| 2 |  | In a number of places the author talks about sex clubs as a place for hetero sex. I think this may be a little over-simplified as many sex clubs welcome and try to attract bi-women (although sometimes not bi-men), and some may for example go with the specific intent of seeking a bi-woman for a threesome. Whether we were to consider threesomes or woman's same-sex sex as now part of hetero sex is open to debate (although it is an argument that's been made) but recognising that clubs are more welcoming of heteronormative sex/ideals rather than specifically hetero sex/heterosexual people might be a useful distinction to make/discuss and provide context. | I completely agree. In fact, one of the aims of the paper was to challenge and disrupt understandings of sex that inadvertently heteronormalize them by thinking of them as mainly places for swingers. In response and where relevant, I have qualified the use of the term sex club. Rather than a club for heterosexuals, they are clubs that are marketed as places for heterosexual sex. They are certainly more than just places for heterosex and I have tried to bring this out a little more in the discussion part of the article. |
| 3 | 2 | Female glory hole - is this a glory hole that excludes men from using? I.e. being the person giving oral sex to others? Further clarity would be good | Yes – good point. I have described what usually a female glory hole looks like. |
| 4 | 2 | The table and discussion of where users leaving reviews are from, and how it relates to population, is difficult to follow (the table especially). A reorganisation/clearer explanation would be helpful. | Yes – agreed. The table has been amended and reorganized. The narrative relating to the table in the findings has been revised and also how this feeds into the discussion – making closer connections between the data and the argument. |
| 5 | 2 | The discussion sometimes goes a bit far away from the data and discusses surrounding and related ideas before linking it back to the data. I feel that a bit of a reorganisation would help with clarity. | I agree, and I think that this relates to the importance of making the argument clearer. In the paragraphs that have been added, I specifically explore the concept of leisure sex. I have then worked through the article to make reference to leisure sense to build the consistency of the article. In other words, this wasn't just about collecting data on the prevalence of sex clubs, this is about the mainstreaming of sex, and that sex clubs are part of an increasing imbrication of leisure and sex. |
| 6 | 3 | Abstract  Please add a sentence on methodology | This has been completed. |
| 7 | 3 | Abstract  I recommend downsizing the intro and devoting more space to the results of this study and their importance and implications. | Yes, agreed. I have cut down the intro and added more on the findings element of the article. |
| 8 | 3 | I recommend presenting early in the paper the research purpose and research questions of this study and then tailor the rest of the paper accordingly. Currently, the earliest statement that could have been intended as a research purpose appears in the methods. This seems late. | I totally agree with this point. Reading back over the article, the research focus is too implicit. I have used the second paragraph to identify the focus of the questions being asked and the contextualizing the broader project. |
| 9 | 3 | The authors need to discuss the difficulty in defining a 'sex club,' present attempts available in the literature, and operationalize it for the current study. Some characteristics are presented in the methods section, but the explicit operationalization is missing. | I agree with this point. A new third paragraph has been written that identifies the ways that sex club has been used in the literature, the multiple ways that sex club is are described in the popular media and provides a more clear description of the definitional criteria that was operationalized to identify clubs in the UK. |
| 10 | 3 | The authors claim having conducted 18 ethnographies. Later in the paper, they clarify that they mean having visited 18 sex clubs. Moreover, all the results presented in this paper are based on descriptive frequency counts. Hence, this paper does not present an ethnography and the authors should revise their methodological claims. | The references to ethnographic data within the paper have been revised to ensure that readers are clear that the data presented in this paper is directly obtained from the sample. |
| 11 | 3 | I am curious as to why the authors have excluded gay and lesbian communities from this study. Presenting some rationale would be helpful. | Yes – absolutely. The article now includes a rationale for the choice of clubs that market themselves to heterosexuals. |
| 12 | 3 | I recommend the authors to engage more in-depth and include relevant methodological literature on sex research in general and on scraping the profile data from the Internet without informed consent. | The issue of consent and data scraping from the internet is a really interesting area to explore. There appear to be conflicting positions on this, and I have updated the methodology to reflect this. Whilst there isn't the space to go into methodological depth on the literature on sex research – there may well be the scope for a separate research paper on this – I have cited research in the methodology that has relevance to this article and how importance is ascribed to the relationship between leisure and sex as an important factor. |
| 13 | 3 | Although this is beyond the scope of this paper, I hope that at some point the authors will actually analyze the content of the posts. | Yes – that is the plan! Although there might need to be some updating given the impact of the pandemic. Sex clubs are being viewed/used/consumed a little differently. |
| 14 | 3 | What technology (if any) was used to scrape the profile data from the internet? What is the potential for human and technical error and how was it mitigated? | I agree this is important to include. A discussion in the methodology has been included that explains the process of the data scraping as well as accounting for any potential technical errors that could have occurred. |
| 15 | 3 | The authors should refer to the tables in the text. | Yes – this has been done! |
| 16 | 3 | The authors need to introduce relevant transitions between the sub-sections to avoid chopped presentations. | This is a good point, especially as one of the discussion paragraphs didn't seem to smoothly progress from one to the other. This has now been re-ordered. |
| 17 | 3 | In the limitations, the authors admit that the results are reflective of the sample of people who left reviews. However, throughout the paper, and even in the abstract, the authors' claims repeatedly make an impression that the results are intended to represent the public attending sex clubs, in general. As the authors admit later, these two populations are different. Hence, this misleading issue should be remedied throughout the paper. | Good point, and sometimes, when writing about people attending clubs, you almost take the caveat 'and leaving reviews' for granted. In response, any insinuation of conflating data collected from people attending clubs and leaving reviews and the general public has been removed. |
| 18 | 3 | The results are valuable but purely descriptive. I recommend the authors to interrogate their data more critically and to run some inferential statistical tests to establish whether the differences between the groups that they discuss are statistically significant. | Whilst I agree that some modelling would be helpful, I am not sure that this is the article that does this. The aim of the paper is really to get the message out there that there is an area of leisure sex that has been under-researched. I just want to help put the sex clubs that are marketed to heterosexuals into the academic line of sight. A more nuanced statistical engagement with the data will be done later. I think this is a really valid point, but one for a paper that goes beyond introducing the issues. |
| 19 | 3 | The authors repeated use the words like 'significant' or 'correlation' but present no statistical results to support these claims. Either such results should be presented (which is a recommended course of action) or the authors should choose an appropriate word without statistical connotations. | Yes – a really good point. As I think the paper would be doing too much all at once with the inclusion of statistical inferences, I have tried to remove all of the terms throughout the article that would imply a statistical approach to the data. |
| 20 | 3 | The authors should explain Table 7 more effectively and also correspond it with the provided narrative. There seemed to be discrepancies and I felt somewhat lost at times. | As pointed out above – I agree. The table has been amended and reorganized. The narrative relating to the table in the findings has been revised and also how this feeds into the discussion – making closer connections between the data and the argument. |
| 21 | 3 | Also, where did the authors obtain the national estimates for the sexual activities? Either it is another instance of inaccurate wording, or a proper presentation of the source is missing. | Given that there is no definitive research in the field, I have changed the sentence to – 'Estimates in the media have suggested that…" |
| 22 | 2 | Acronyms: (1) the authors need to define/present their meaning before using the acronyms; (2) the authors appear to use CNM and NCM interchangeably, probably a typo. | Yes – this has been done! |
| 23 | 3 | (Theoretical) Important but needs more work. | This has been worked on, and the article aligns itself much more with the theoretical approaches associated with leisure sex. |
| 24 | 3 | Good, but additional work on depth would be helpful. | Where possible and with an eye on words, a more in-depth engagement has been pursued. |
